# Relationship between Serum Uric Acid Levels and the Severity of Pulmonary Hypertension

**Published:** 2017-06

**Authors:** Seyyed Reza Seyyedi, Majid Malekmohammad, Mandana Chitsazan, Neda Behzadnia, Makan Sadr, Seyed Mohammadreza Hashemian, Babak Sharif-Kashani

**Affiliations:** 1 Lung Transplantation Research Center, Department of Cardiology, National Research Institute of Tuberculosis and Lung Diseases (NRITLD), Shahid Beheshti University of Medical Sciences, Tehran, Iran,; 2 Tracheal Diseases Research Center, NRITLD, Shahid Beheshti University of Medical Sciences, Tehran, Iran,; 3 Virology Research Center, NRITLD, Shahid Beheshti University of Medical Sciences, Tehran, Iran,; 4 Chronic Respiratory Diseases Research Center, NRITLD, Shahid Beheshti University of Medical Sciences, Tehran, Iran

**Keywords:** Uric acid, Severity, Pulmonary hypertension

## Abstract

**Background:**

Right heart catheterization is the gold standard test for diagnosis and clinical assessment of the patients with pulmonary hypertension (PH). In recent years, the usefulness of cheaper and non-invasive tests in the follow-up of PH patients is being studied. The aim of the present study was to evaluate the relationship between serum uric acid level and severity of pulmonary hypertension in PH patients.

**Materials and Methods:**

In a cross-sectional study, serum uric acid was measured in 110 patients with PH (63 women; mean age [±SD] was 52.83±17.88 years). Pulmonary arterial pressure and severity of right ventricular dysfunction were assessed using RHC and echocardiography, respectively.

**Results:**

Serum uric acid was higher in PH patients with severe RV dysfunction, compared to mild and moderate dysfunction (7.8mg/dl [IQR: 5.8–9.2] in severe dysfunction, versus 4.7 mg/dl [3.87–5.82] in mild dysfunction and 5 mg/dl [3.5–6.95] in moderate dysfunction. Serum uric acid was significantly correlated with pulmonary artery systolic pressure (r=0.51, P<0.001). Serum uric acid level also had a significant positive correlation with the World Health Organization functional class of the patients (r=0.49, P<0.001). Serum uric acid level greater than 5.7 mg/dl was found to be the most sensitive and specific points for predicting severe RV dysfunction in PH patients (sensitivity 76.6%, specificity 71.4%; AUC=0.79, P<0.001) .

**Conclusion:**

Serum uric acid is correlated with the severity of symptoms and RV dysfunction in patients with pulmonary hypertension. Further studies are recommended with larger sample size in this regard.

## INTRODUCTION

Pulmonary hypertension (PH) consists of a group of disorders characterized with proliferative and obstructive remodeling of pulmonary vessels, leading to progressive increase in pulmonary vascular resistance (PVR) and pulmonary arterial pressure (PAP). The increased right ventricular afterload ultimately results in right heart failure and death(1). It is a fatal disease with an estimated mortality rate of 6.5 per 100,000 persons (2). PH is defined as resting mean pulmonary arterial pressure (mPAP) greater than 25 mmHg, measured by right heart catheterization (RHC) (3). RHC is the gold standard for the diagnosis of PH and hemodynamics assessed during RHC are established prognostic factors, both at the diagnosis and during the patient follow-up(4). However, RHC is an expensive and invasive procedure and it is recommended to be performed in experienced centers to avoid serious complications (5). In recent years, the utility of noninvasive procedures in the clinical assessment of the patients during follow-up have been studied extensively and they are shown to provide important information about the severity of the disease and survival of the patients (6).

Measurement of serum uric acid, as a marker of decreased cardiac output and tissue hypoxia, has been adopted in the clinical assessment of pulmonary hypertension(4). Serum uric acid is a non-invasive and inexpensive test that is widely available in all centers. Serum uric acid level has been shown to be increased in right or left heart failure (7). Hyperuricemia is shown to have a strong correlation with the severity of symptoms and functional capacity of the patients with heart failure (8). In a study by Nagaya et al, serum uric acid was significantly higher in patients with PH and it was independently related to mortality (9). In another study, hyperuricemia was common in patients with severe PH and there was a positive correlation between serum uric acid level and right atrial pressure elevation in PH patients (10).

In this study, we aimed to evaluate the relationships between serum uric acid level and severity of symptoms and right ventricular (RV) dysfunction in patients with confirmed PH patients.

## MATERIALS AND METHODS

This is an observational cross-sectional study conducted in the expert PH clinic of Masih Daneshvari Hospital, a tertiary center in Tehran, Iran. All consecutive patients with established diagnosis of PH being treated at the clinic were included in the study between January 2012 and April 2017. Patients were excluded if they were taking allopurinol or a uricosuric agent; PH was secondary to hypoxic pulmonary diseases such as chronic obstructive pulmonary disease (COPD), bronchiectasis, arteriovenous malformation (AVM); there was a coexisting renal or hepatic disease; or when serum creatinine was above 1.5 mg/dl in men and 1.2 mg/dl in women.

Data regarding basic demographics, physical examination, WHO functional class (11), basic laboratory data, serum uric acid levels, transthoracic echocardiography and right heart catheterization (RHC) were gathered in all patients. Fasting venous blood was sampled to measure uric acid levels. Serum uric acid levels were determined using the uricase-peroxidase method. Transthoracic Echocardiography (TTE) was performed in all patients with Vivid 7 Dimension echocardiography machine (GE Healthcare, Horten, Norway) using a 4 MHz probe. Right ventricular function was assessed by an experienced cardiologist based on visual assessment of RV free wall motion, septal motion, and RV size in the parasternal long-axis, apical four chamber and substernal views and also quantitative measurement of the tricuspid annular plane systolic excursion (TAPSE), RV fractional area change (RVFAC), and peak systolic myocardial velocity by Doppler tissue imaging (RVSm)(12). Accordingly, RV dysfunction was subjectively categorized as mild, moderate or severe dysfunction. RHC was performed in the supine position from the right femoral vein using a swan-Ganz catheter. Pulmonary artery systolic pressure (PASP) was measured in all patients.

The study protocol was approved by the Institutional Review Board of the Research Institute of Tuberculosis and Lung Diseases and written informed consent was obtained from all participants.

### Statistical analysis

Non-normally distributed data were compared between groups using Kruskall-Wallis test. The Chi square test was used for categorical data. Spearman rho correlation was used to investigate the correlation between uric acid level and variables. Mann-Whitney U test was used to compare the mean serum uric acid level in patients with and without electrocardiographic signs of RV strain. We used Receiver operator characteristic curve (ROC) to estimate the threshold values of serum uric acid to predict severe PH. The area under the ROC curve (AUC) and the corresponding standard error were calculated. The data were analyzed using SPSS for Windows version 18 (Chicago, IL, USA). All reported p-values are two-tailed, and p-values of less than 0.05 were considered statistically significant.

## RESULTS

One hundred and ten patients, including 47 (42.7%) males and 63(57.3%) females were enrolled in the study. The mean age was 52.83±17.88 years. Most of the patients (40 patients; 36.4%) were categorized as WHO functional class IV. Twenty nine patients (26.4%) were in WHO class III. Twenty five patients (22.7%) and 16 patients (14.5%) were in WHO class II and class I, respectively.

The basic characteristics of the patients are presented in table 1. As shown in table 1, patients with severe RV dysfunction were significantly younger than patients with mild and moderate RV dysfunction. Severe RV dysfunction was also more common in women compared to men, while patients with mild RV dysfunction were more commonly men (P=0.001).

**Table 1.** Baseline characteristics of the patients

**Variable**	**Mild RV dysfunction (n=42)**	**Moderate RV dysfunction (n=21)**	**Severe RV dysfunction (n=47)**	**P value**
Age, years	62.86±13.34	58.19±15.60	41.47±16	<0.001
Gender, n (%)				0.001
Male	26(61.90)	10 (47.60)	11 (23.40)	
Female	16(38.10)	11 (52.40)	36 (76.60)	
WHO FC, n (%)				<0.001
I	16 (38.10)	0 (0)	0	
II	24 (47.10)	1 (4.80)	0	
III	2 (4.80)	18 (85.70)	9 (19.10)	
IV	0 (0)	2 (9.50)	38 (80.90)	
Cause				<0.001
IPAH	7(16.70)	5(23.80)	33(70.20)	
CTEPH	7(16.70)	4(19.05)	9(19.10)	
Connective tissue disease	8(19.05)	3(14.30)	2(4.30)	
Sarcoidosis	9(21.40)	4(19.05)	0	
Asthma	11(26.20)	5(23.80)	0	
PVOD	0	0	2(4.30)	
Thalassemia	0	0	1(2.10)	
Pulmonary artery systolic pressure, mmHg^*^	40 (35–50)	55 (42.50–70)	80 (75–95)	<0.001
Uric acid, mg/dl^*^	4.70 (3.87–5.82)	5 (3.50–6.95)	7.80 (5.80–9.20)	<0.001

* Data expressed as median (IQR)

WHO FC: WHO functional class; IPAH: Idiopathic Pulmonary Arterial Hypertension; CTEPH: Chronic Thromboembolic Pulmonary Hypertension; PVOD: Pulmonary Veno-Occlusive Disease

**Figure 1.**
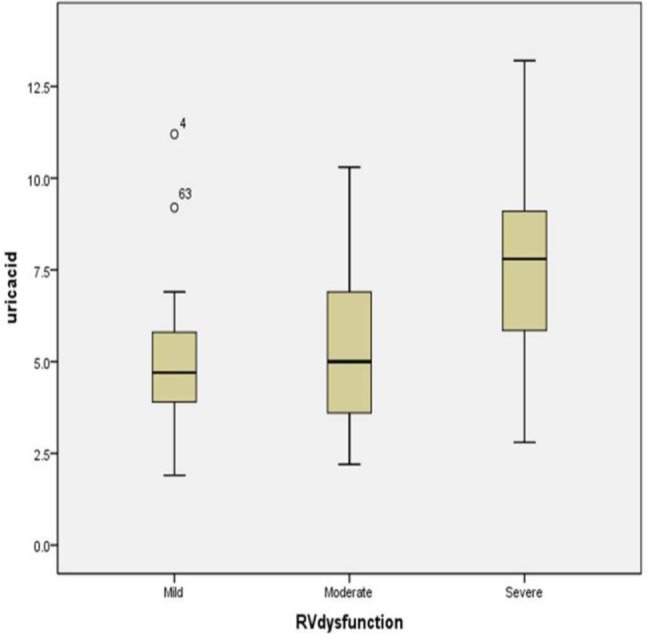
Distribution of uric acid level in pulmonary hypertension patients with mild, moderate and severe RV dysfunction

PASP was significantly higher in patients with severe RV dysfunction (P<0.001). Heart rate was also significantly higher in patients with severe RV dysfunction (P<0.001). Serum uric acid was higher in patients with severe RV dysfunction compared to mild and moderate RV dysfunction and the observed difference was statistically significant (P<0.001) (Figure 1). The difference between mild and moderate RV dysfunction was not significant (P=0.34).

The correlation between the uric acid and age, heart rate, pulmonary artery systolic pressure, and WHO functional class is shown in table 2. Serum uric acid level had a weak negative correlation with age (r=−0.22, P=0.024). However, after controlling for severe RV dysfunction, the correlation between age and serum uric acid was not significant anymore(r=0.16, P=0.108).

**Table 2.** Correlations between uric acid level and other variables

**Variable**	**Correlation coefficient**	**P value**
Age	−0.22	0.024
Heart rate	0.05	0.607
PASP	0.51	<0.001
WHO FC	0.49	<0.001

PASP: Pulmonary artery systolic pressure; WHO FC: WHO functional class

**Figure 2.**
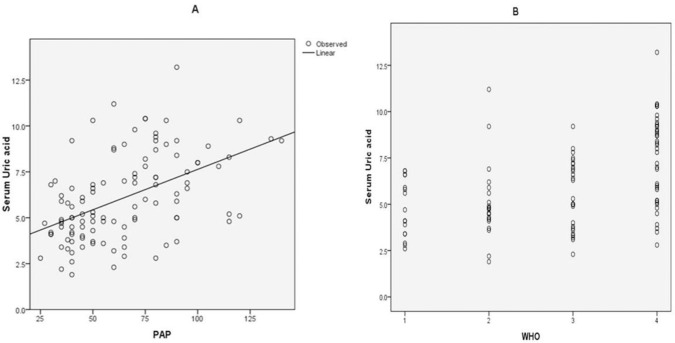
Correlation between pulmonary artery systolic pressure (PASP) and uric acid level (A) and Correlation between WHO functional class and uric acid level (B) in patients with pulmonary hypertension.

Uric acid level was not significantly correlated with heart rate (r=0.05, P=0.607). Serum uric acid was significantly correlated with PASP (r=0.51, P <0.001), indicating that higher uric acid level was associated with higher PASP (Figure 2.A).Serum uric acid level also had a significant positive correlation with the WHO functional class (r=0.49, P <0.001), meaning that higher uric acid level was associated with a worse WHO functional class (Figure 2.B).

**Figure 3.**
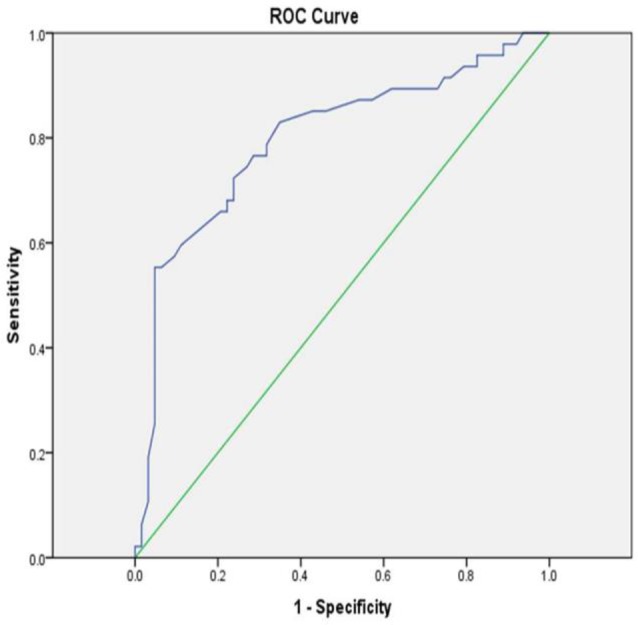
ROC of serum uric acid level for prediction of severe RV dysfunction in PH patients

Optimum level of uric acid for the prediction of severe PH was 5.7 mg/dl (sensitivity and specificity of 76.6% and 71.4%, respectively) (AUC=0.798, 95% CI: 0.709− 0.886; P<0.001) (Figure 3).

## DISCUSSION

The main findings of our study were that PH patients with severe RV dysfunction tend to be younger females with higher heart rate, PASP and uric acid levels compared to patients with mild and moderate RV dysfunction; and Serum uric acid level was significantly correlated with PASP and WHO functional class. In a study by Zhang et al, conducted in 86 patients with IPAH, there was a significant positive correlation between serum uric acid levels and PASP(13). In their study, the correlation between serum uric acid levels and heart rate was not statistically significant. However, the correlation between serum uric acid levels and NYHA functional class was significant (r=0.41, p<0.01) and similar to our results (r=0.49, p<0.001)

Moreover, in our study uric acid level was negatively correlated with age, i.e. younger patients had higher uric acid levels. It was in contrast with the evidence that uric acid level increases with age (14). The authors believe that this discrepancy could be explained by the fact that in our patients uric acid levels were higher in patients with severe RV dysfunction and most of the patients with severe RV dysfunction were younger females (with PH secondary to IPAH). The confounding effect of severe RV dysfunction in the correlation between age and uric acid was confirmed after adjustment.

The exact mechanism of increased uric acid level in patients with heart failure is unclear. Uric acid is the final product in the metabolism of purines. Hypoxia impairs the formation of adenosine triphosphate (ATP), and degradation of ATP to adenosine diphosphate and monophosphate occurs. This process leads to increased purine metabolites, with the end result of overproduction of uric acid (15). On the other hand, low cardiac output and the resultant decreased glomerular filtration could possibly impair renal excretion of uric acid (7).

Serum uric acid level is dependent on several other factors, such as age, gender, diuretic use and renal function. The main limitation of our study was that all of our patients were taking diuretics. However, the authors believe that higher uric acid level could not be merely due to diuretic use, since the renal function of all patients was normal. We suggest that further studies with long-term follow-up of the patients and measurement of the serum uric acid level changes after treatment should be performed to better determine the role of hyperuricemia in survival of PH patients.

In conclusion, serum uric acid level is significantly correlated with severity of symptoms and RV dysfunction in PA patients. Therefore it can be used as a non-invasive test in the follow-up of the PH patients. Increased uric acid level in PH patients indicates worsening of the RV function and may warrant considering modification of the treatment regimen and optimally referring the patient to an expert PH center. Further studies with larger sample sizes are also recommended in this regard.

## References

[1] Howard LS Prognostic factors in pulmonary arterial hypertension: assessing the course of the disease. Eur Respir Rev 2011;20:236–42. 22130816 10.1183/09059180.00006711 PMC9487744

[2] George MG Schieb LJ Ayala C Talwalkar A Levant S Pulmonary hypertension surveillance: United States, 2001 to 2010. Chest 2014;146:476–495. 24700091 10.1378/chest.14-0527 PMC4122278

[3] Hoeper MM Bogaard HJ Condliffe R Frantz R Khanna D Kurzyna M Definitions and diagnosis of pulmonary hypertension. J Am Coll Cardiol 2013; 62:D42–50. 24355641 10.1016/j.jacc.2013.10.032

[4] Galiè N Humbert M Vachiery JL Gibbs S Lang I Torbicki A 2015 ESC/ERS Guidelines for the diagnosis and treatment of pulmonary hypertension: The Joint Task Force for the Diagnosis and Treatment of Pulmonary Hypertension of the European Society of Cardiology (ESC) and the European Respiratory Society (ERS): Endorsed by: Association for European Paediatric and Congenital Cardiology (AEPC), International Society for Heart and Lung Transplantation (ISHLT). Eur Heart J 2016;37:67–119. 26320113 10.1093/eurheartj/ehv317

[5] Hoeper MM Lee SH Voswinckel R Palazzini M Jais X Marinelli A Complications of right heart catheterization procedures in patients with pulmonary hypertension in experienced centers. J Am Coll Cardiol 2006;48:2546–52. 17174196 10.1016/j.jacc.2006.07.061

[6] Foris V Kovacs G Tscherner M Olschewski A Olschewski H Biomarkers in pulmonary hypertension: what do we know? Chest 2013;144:274–283. 23880678 10.1378/chest.12-1246

[7] Hoeper MM Hohlfeld JM Fabel H Hyperuricaemia in patients with right or left heart failure. Eur Respir J 1999;13:682–5. 10232447 10.1183/09031936.99.13368299

[8] Leyva F Anker S Swan JW Godsland IF Wingrove CS Chua TP Serum uric acid as an index of impaired oxidative metabolism in chronic heart failure. Eur Heart J 1997;18:858–65. 9152657 10.1093/oxfordjournals.eurheartj.a015352

[9] Nagaya N Uematsu M Satoh T Kyotani S Sakamaki F Nakanishi N Serum uric acid levels correlate with the severity and the mortality of primary pulmonary hypertension. Am J Respir Crit Care Med 1999;160:487–92. 10430718 10.1164/ajrccm.160.2.9812078

[10] Voelkel MA Wynne KM Badesch DB Groves BM Voelkel NF Hyperuricemia in severe pulmonary hypertension. Chest 2000;117:19–24. 10631193 10.1378/chest.117.1.19

[11] Rich S Executive summary from the World Symposium on Primary Pulmonary Hypertension 1998, Evian, France, September 6–10, 1998, cosponsored by the World Health Organization Retrieved April. 2000;14.

[12] Rudski LG Lai WW Afilalo J Hua L Handschumacher MD Chandrasekaran K Guidelines for the echocardiographic assessment of the right heart in adults: a report from the American Society of Echocardiography endorsed by the European Association of Echocardiography, a registered branch of the European Society of Cardiology, and the Canadian Society of Echocardiography. J Am Soc Echocardiogr 2010;23:685–713; quiz 786–8. 20620859 10.1016/j.echo.2010.05.010

[13] Zhang CY Ma LL Wang LX Relationship between serum uric acid levels and ventricular function in patients with idiopathic pulmonary hypertension. Exp Clin Cardiol 2013;18:e37–9. 24294046 PMC3716500

[14] Kuzuya M Ando F Iguchi A Shimokata H Effect of aging on serum uric acid levels: longitudinal changes in a large Japanese population group. J Gerontol A Biol Sci Med Sci 2002;57:M660–4. 12242321 10.1093/gerona/57.10.m660

[15] Saito H Nishimura M Shibuya E Makita H Tsujino I Miyamoto K Tissue hypoxia in sleep apnea syndrome assessed by uric acid and adenosine. Chest 2002;122:1686–94. 12426272 10.1378/chest.122.5.1686

